# Amniotic fluid exerts a neurotrophic influence on fetal neurodevelopment via the ERK/GSK-3 pathway

**DOI:** 10.1186/s40659-015-0029-4

**Published:** 2015-08-05

**Authors:** Yongwoo Jang, Eun-Kyung Kim, Won-Sik Shim, Ki-Min Song, Sung Min Kim

**Affiliations:** College of Pharmacy, Seoul National University, Seoul, 151-742 South Korea; McLean Hospital, Harvard Medical School, Belmont, MA 02478 USA; College of Pharmacy, Gachon University, Incheon, 406-840 South Korea; Department of Health Sciences, Hanyang University, Seoul, 133-791 South Korea; Department of Physical Education, College of Performing Arts and Sport, Hanyang University, Seoul, 133-791 South Korea

**Keywords:** Amniotic fluid, Extracellular signal-regulated kinase (ERK), Glycogen synthase kinase-3 (GSK-3), Neural clustering, Neurosphere, Fetal development

## Abstract

**Background:**

The fetus is surrounded by the amniotic fluid (AF) contained by the amniotic sac of the pregnant female. The AF is directly conveyed to the fetus during pregnancy. Although AF has recently been reported as an untapped resource containing various substances, it remains unclear whether the AF could influence fetal neurodevelopment.

**Results:**

We used AF that was extracted from embryos at 16 days in pregnant SD rat and exposed the AF to the neural cells derived from the embryos of same rat. We found that the treatment of AF to cortical neurons increased the phosphorylation in ERK1/2 that is necessary for fetal neurodevelopment, which was inhibited by the treatment of MEK inhibitors. Moreover, we found the subsequent inhibition of glycogen synthase kinase-3 (GSK-3), which is an important determinant of cell fate in neural cells. Indeed, AF increased the neural clustering of cortical neurons, which revealed that the clustered cells were proliferating neural progenitor cells. Accordingly, we confirmed the ability of AF to increase the neural progenitor cells through neurosphere formation. Furthermore, we showed that the ERK/GSK-3 pathway was involved in AF-mediated neurosphere enlargement.

**Conclusions:**

Although the placenta mainly supplies oxygenated blood, nutrient substances for fetal development, these findings further suggest that circulating-AF into the fetus could affect fetal neurodevelopment via MAP kinases-derived GSK-3 pathway during pregnancy. Moreover, we suggest that AF could be utilized as a valuable resource in the field of regenerative medicine.

## Background

In placental mammals, the placenta is physically connected with the developing embryos or fetus through the umbilical cord, which supplies oxygenated blood and nutrient substances for fetal development. Moreover, the amniotic fluid (AF), enveloped by the amniotic sac of a pregnant female, is a water-like fluid that is inhaled and exhaled by the fetus [1]. Although AF contains many nutrients and potentially deleterious materials, its effects on fetal neurodevelopment are elusive.

Because the fetus is surrounded by the water-like AF, it was originally considered to function as a shock absorber to protect against external impacts. However, it has been recently reported that AF contains various proteins, carbohydrates, lipids, and urea, all of which circulated into fetus [1–3]. During the growth of the fetus, the volume of the AF increases faster than embryonic size, and is correlated positively with the development of the fetus [1]. The amnion is an active membrane that closely covers the embryo, which metabolically secretes nutritious factors to maintain homeostasis of solutes and water in the AF [4, 5]. In fact, it has been revealed that nerve growth factor (NGF), brain-derived neurotrophic factor (BDNF), and neurotrophin-3 (NT-3) are present in human AF [6]. Moreover, several factors in AF are also believed to be conveyed directly to the fetus, in response to physiological changes in the maternal body during pregnancy [7]. In pregnancy, maternal stress or anxiety increases a stress hormone, cortisol levels in maternal plasma, which are also found correlatively in AF [8]. It has also been reported that maternal obesity increases the level of inflammatory cytokines, such as TNF-α and interleukin-8 in AF [9]. Taken together, these factors could potentially influence the development of embryonic neurons through neurotropic cascade signaling.

In developing neurons, the mitogen-activated protein (MAP) kinases pathway is a multi-functional signaling cascade that regulates neuronal proliferation, differentiation, and apoptosis responding to growth factors, neurotransmitters, neurotrophins, and hormones. These extracellular stimuli typically induce the activation of tyrosine receptor kinase receptors (TRKs) or G-protein-coupled receptors (GRCRs), which subsequently triggers the MAP kinases pathway. The MAP kinases have largely been classified into three major groups: extracellular signal-regulated kinase (ERK), c-jun N-terminal kinase (JNK), and p38 MAP kinase [10]. In particular, ERK is a versatile protein kinase that has been implicated in the proliferation and differentiation of neural progenitor cells. Thus, we examined whether AF stimulates or inhibits the MAP kinases pathway in differentiated neurons and neural progenitor cells, and then investigated the underlying cellular signaling and AF’s effects in this study.

## Results

### AF activated ERK, time- and dose-dependently, in cortical neurons

**Fig. 1:**
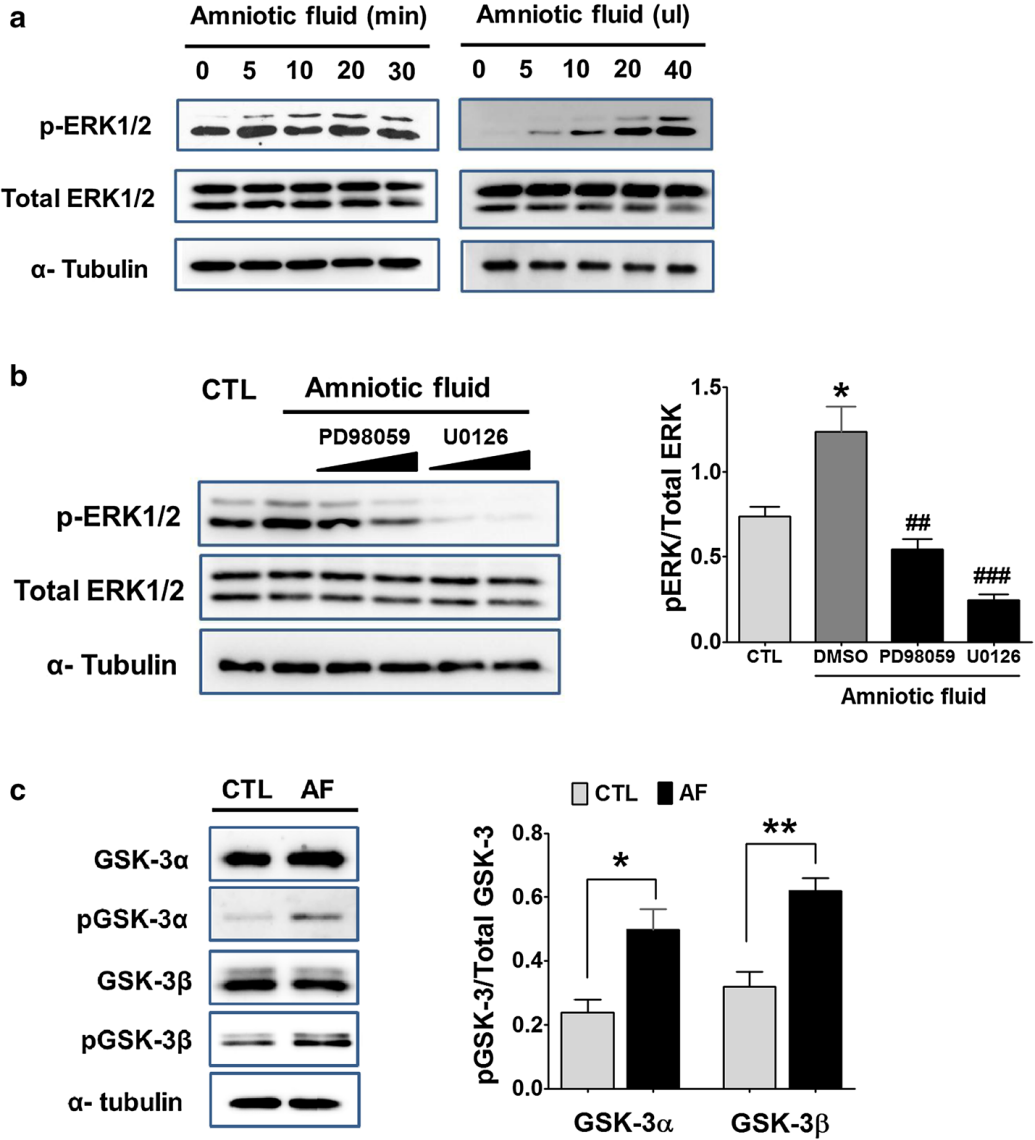
After treatment of cortical neurons with AF, the phosphorylation of ERK was determined by Western blotting. **a** Phosphorylation of ERK was increased by AF treatment in a time- (*left*) and dose-dependent manner (*right*). **b** ERK phosphorylation was reduced by treatment with MEK inhibitors, PD98059 and U0126. In cortical neurons, treatment with AF (20 μL/mL) AF for 20 min showed a significant increase in the ERK phosphorylation whereas the application of PD98059 or U0126 reduced its phosphorylation. *p < 0.05, versus CTL cells (CTL); ^##^p < 0.01, versus AF and DMSO treated cells (DMSO); ^###^p < 0.001, versus DMSO-treated cells (DMSO). **c** After treatment of cortical neurons with AF, the phosphorylation of GSK-3α and 3β was determined by Western blotting. Phosphorylation of GSK-3 was increased by the treatment of AF. A representative blot (*left*) and statistical bar graph (*right*). *p < 0.05, **p < 0.01, versus CTL cells (CTL).

To investigate the influence of AF on fetal brain development, we first determined the effect of AF on primary cultured cortical neurons. Because the MAP kinases pathway is essential in the development of neurons [10], we investigated the phosphorylation of ERK, a key kinase in the MAP kinases pathway [11], after the treatment of cortical neurons with AF. As can be seen in Fig. 1a, the application of AF to cortical neurons increased the phosphorylation of ERK1/2 markedly, in a time- and dose-dependent manner. Next, we determined whether this ERK1/2 phosphorylation was mediated by MEK1/2, up-stream of ERK1/2. When MEK1/2 inhibitors (PD98059, U0129) were added with AF, ERK1/2 phosphorylation was inhibited significantly in the cortical neurons (PD98059, 0.54 ± 0.08; UO126, 0.24 ± 0.04, n = 4) (Fig. 1b). Indeed, we could detect the level of growth factor like NGF in AF (data not shown). Thus, these results showed that AF activated the MAP kinases pathway in primary cortical neurons. Furthermore, we questioned the down-stream signal of ERK1/2, activated by AF. Glycogen synthase kinase-3 (GSK-3) is a serine/threonine protein kinase, composed of GSK-3α and GSK-3β. Its activity is inhibited by phosphorylation on serine residues of GSK-3α (Ser 21) and GSK-3β (Ser 9), which is an important determinant of cell fate in neural stem or progenitor cells [12]. Indeed, it has been reported that GSK-3 inhibitors, such as lithium (Li^+^), can regulate the proliferation of neural progenitors and neuronal growth through the ERK pathway [13, 14]. Thus, we next focused on GSK-3 activity as a down-stream signal of the ERK1/2-mediated MAP kinase pathway in response to AF. As can be seen in Fig. 1c, the treatment of cortical neurons with AF showed significantly increased phosphorylation of GSK-3α and GSK-3β; that is, the inhibitory form (Student’s *t*-test, *<0.05, **<0.01, n = 4). This result indicates that AF inactivated GSK-3α and GSK-3β through the MAP kinase pathway.

### AF induced the formation of cell clustering in cortical neurons

**Fig. 2:**
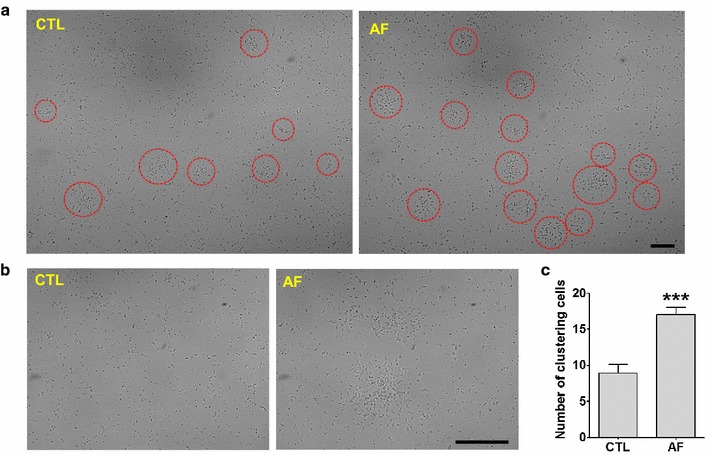
Functional activity of AF in differentiated neurons. **a** Images at 5 days after treatment with vehicle (*left*) or AF (*right*) in the cortical neurons. The *red circle* indicates the clustered neural cells. AF-treated cortical neurons showed increased formation of neural clustering than control cells. *Scale bar* indicates 100 μm. **b** These pictures represent a magnification of the formation of neural clustering. *Scale bar* indicates 100 μm. **c** AF-treated cortical neurons showed increased formation of clustering cells. ***p < 0.001, versus CTL cells (CTL).

The numerous studies support the concept that activity of GSK-3α and GSK-3β is involved in cell fate, including in neuronal proliferation and differentiation [12–14]. So, we next observed phenotypic changes in cultured cortical neurons after application of AF. Interestingly, AF significantly increased the formation of cell clustering when compared to that of control cells (Student’s *t*-test, ***<0.001, n = 10) (Fig. 2c). Interestingly, since the spherical shape of cluster is reminiscent of neural progenitor or stem cells (Fig. 2a, b) [15], we then checked the properties of these cell clusters.

**Fig. 3:**
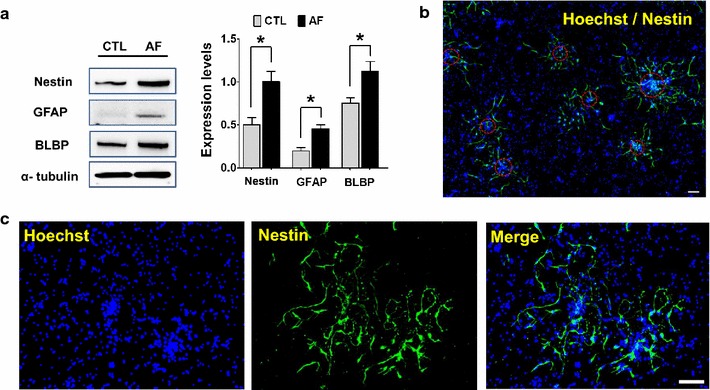
Identification of clustering cells. **a** Western blot from the cortical neurons cultured for 5 days after treatment with vehicle or AF. AF-treated cells showed a significant increase in neural stem or progenitor cell markers such as Nestin, GFAP, and BLBP. *p < 0.05, versus CTL cells (CTL). **b** The nuclei of cells were stained with Hoechst 33342 (*blue*). The *red circles* indicate the clustered cells, which are focally merged with Nestin (*green*). *Scale bar* indicates 60 μm. **c** These pictures represent a magnification of the staining with Nestin. Nestin-positive neural stem or progenitor cells were observed in the clustered cells. *Scale bar* indicates 60 μm.

**Fig. 4:**
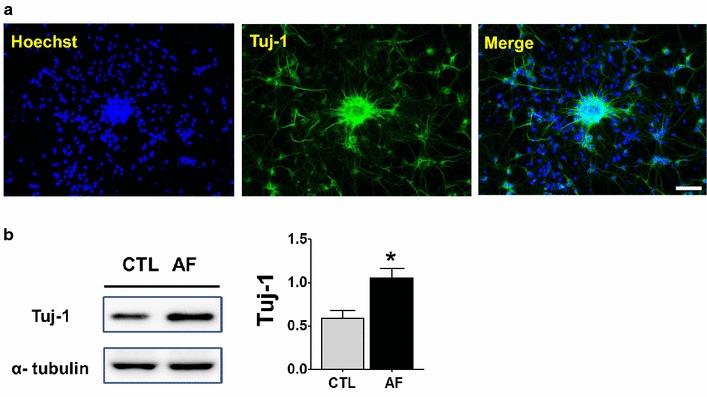
Promotion of neural cell lineage in response to AF. **a** The nuclei of cells were stained with Hoechst 33342 (*blue*). The clustered cells were stained with immature neuronal marker, Tuj-1 (*green*). *Scale bar* indicates 60 μm. **b** Western blot from the cortical neurons cultured for 5 days after treatment with vehicle or AF. AF-treated cells showed a significant increase in immature neuronal marker, Tuj-1. *p < 0.05, versus CTL cells (CTL).

As expected, it was found that the expression of neural progenitor cell markers, such as Nestin, GFAP, and BLBP was increased by Western blot (Student’s *t*-test, *<0.05, n = 4) (Fig. 3a) [16–18]. Moreover, immunostaining with Nestin also revealed that the clustered cells are likely to be neural progenitor cells (Fig. 3b, c). To further clarify whether the clustered cell are indeed from the neural lineage, cells were further stained with an immature neuronal marker, Tuj-1 (Neuron-specific class III beta-tubulin). As can be seen in Fig. 4a, intense immune-reactivity against Tuj-1 was evident in migrating cells spreading out from the clustered sphere. As expected, the quantity of neural cells was significantly increased in cortical cells treated with AF (Student’s *t*-test, *<0.05, n = 4) (Fig. 4b). Taken together, AF improves neural progenitor cell pool, inducing neural clustering formations.

### AF increased the formation of neurospheres

**Fig. 5:**
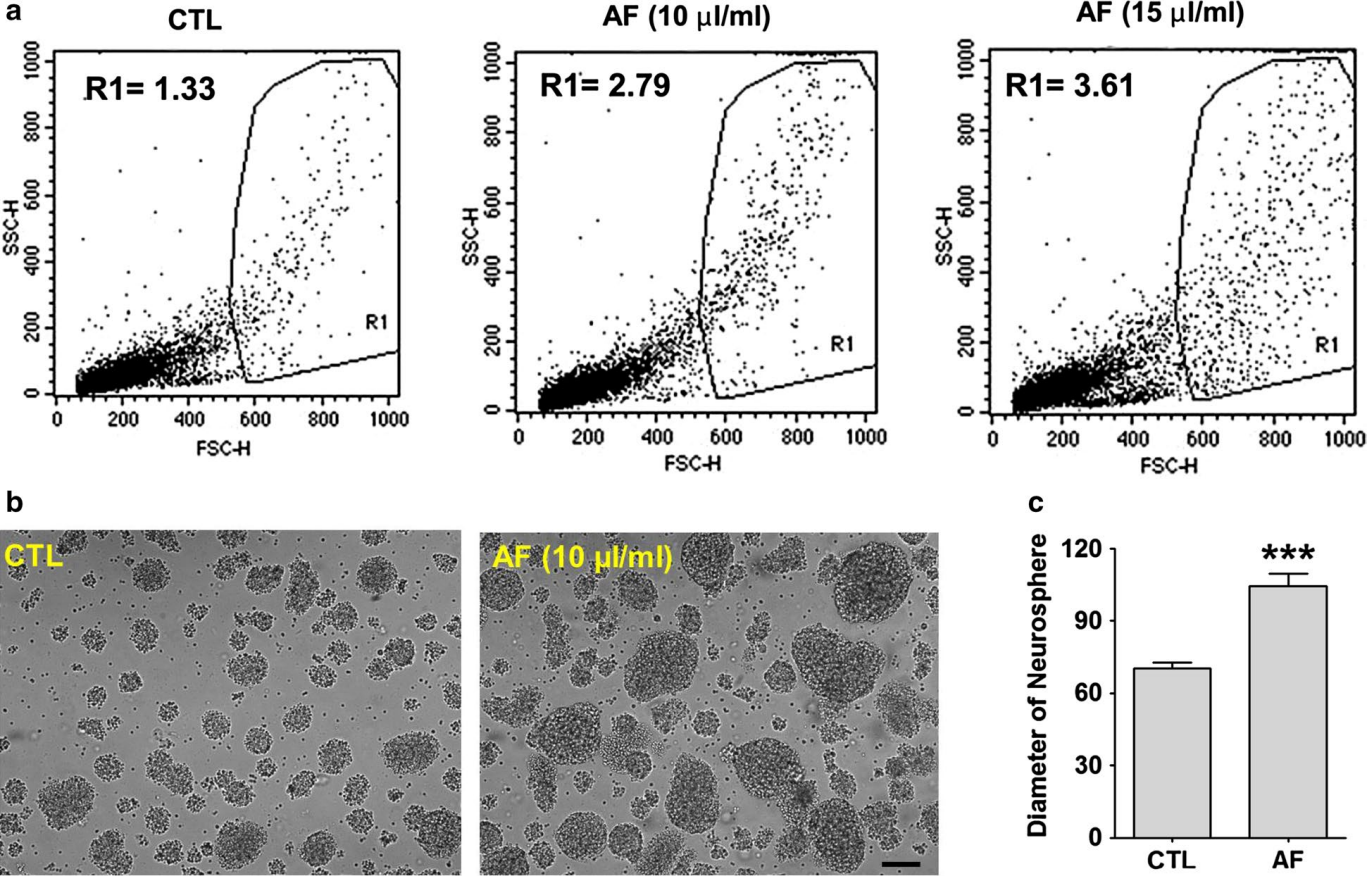
Formation of neurospheres in response to AF. **a** The comparative sizes of neurospheres were measured by flow cytometry. FSC-H indicates the height of forward scatter (FSC), which is proportional to cell size. The R1 area represents enlarged neurosphere (CTL, 1.33; 10 μL/mL AF, 2.79; 15 μL/mL AF, 3.61). **b** Representative pictures at 5 days after treatment with vehicle or AF. **c** Statistical analysis on the formation of neurosphere. The AF-treated cells showed a significant increase in the diameter of neurosphere comparing to control cells. ***p < 0.001 versus CTL cells (CTL). *Scale bar* indicates 100 μm.

To further confirm the formation of neural clustering, we compared the formation of neurospheres, composed of free-floating clusters of neural stem or progenitor cells. When cortical cells isolated from embryonic cortex are plated on uncoated plastic plate in N2-supplemented medium containing bFGF and EGF, the proliferating cells form free-floating clusters with properties of neural stem or progenitor cells [19]. First, flow cytometric analysis was performed to analyze a relative size of neurospheres with or without AF. A higher frequency of enlarged neurospheres was observed in R1 area with increasing concentrations of AF (R1; CTL, 1.33; 10 μL/mL AF, 2.79; 15 μL/mL AF, 3.61; Fig. 5a). To further confirm the size difference, we measured the diameter of the neurospheres. As can be seen in Fig. 5b, we confirmed that AF enlarged the formation of neurospheres compared with the control sphere (Student’s *t*-test, p < 0.001; Fig. 5c). Thus, AF has the ability to cause the proliferation of neural stem cells.

### AF-induced neurosphere enlargement was dependent on the MAP kinases and GSK-3 pathway

**Fig. 6:**
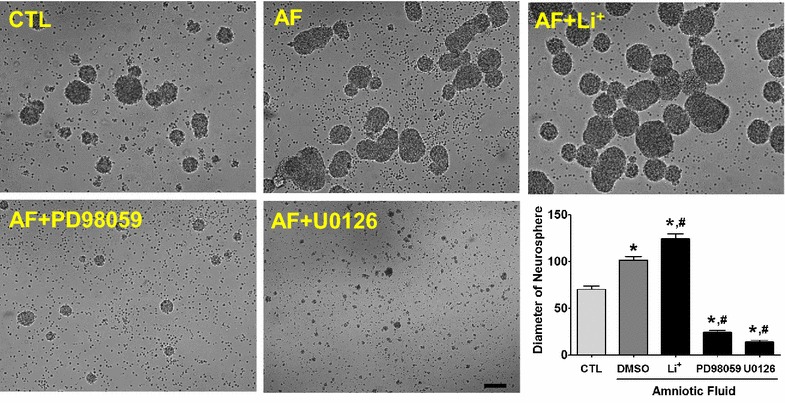
MAP kinases pathway in the formation of neurospheres. AF-treated cells showed increased enlargement of neurospheres compared with control cells. In the presence of AF, application of the GSK-3 inhibitor, Li^+^, caused enlarged formation of neurospheres versus the control and AF alone. However, treatment with MEK inhibitors, such as PD98059 or U0126, inhibited the formation of neurosphere. *p < 0.05, versus CTL cells (CTL); ^#^p < 0.05, versus vehicle (DMSO)-treated cells (DMSO), one way ANOVA, Tukey’s post hoc test. *Scale bar* indicates 100 μm.

We next investigated whether the AF-induced MAP kinases and GSK-3 pathway was involved in neurosphere enlargement. So, we compared the formation of neurospheres after pharmacological perturbation. As can be seen in Fig. 6, treatment with AF caused enlarged neurosphere formation versus the control neurosphere, whereas co-treatment plus PD98059 or U0126 with AF inhibited the formation of neurospheres, indicating that AF-induced neurosphere enlargement was dependent on the MAP kinases pathway. Moreover, we confirmed a possible contribution to GSK-3 on neurosphere formation. According to previous reports, GSK-3 is known to be inhibited by Li^+^, augmenting the phosphorylation of inhibitory serine residues on GSK-3 [20]. Thus, we treated with the GSK-3 inhibitor, Li^+^, during the formation of neurospheres. As can be seen in Fig. 6, the application of Li^+^ in the presence of AF showed a greater increase in the diameter of the neurosphere than the control and AF. Thus, the inhibition of GSK-3 is involved in the formation of neurospheres. Taken together, AF enlarged the neurospheres formed, involving MAP kinases and the GSK-3 pathway.

## Discussion

The present study shows that AF exhibits neurotrophic effects on fetal neurodevelopment during pregnancy. The treatment of embryonic cortical neurons with AF induced the MAP kinases pathway markedly, a key signaling pathway in neural development. Subsequently, we found the AF-induced MAP kinases activation has a suppressive effect on GSK-3 activity in cortical neurons. After the application of AF to cultured cortical neurons, we observed an increased neural clustering that resembled neural stem or progenitor cells. Indeed, we further showed that the AF-derived MAP Kinases/GSK-3 pathway was implicated in the proliferation of neural progenitor cells.

Recently, AF has been actively investigated for various functions beyond its role as a shock absorber. Interestingly, amniotic membrane and fluid-derived cells release neurotrophic factors required for neuron survival [4]. In fact, implantation of human amniotic epithelial cells protects against the degeneration of dopaminergic neuron in a rat model of Parkinson’s disease [21]. Moreover, medium conditioned by human amniotic epithelial cells improved the survival of rat retinal ganglion cells [5]. Thus, it seems that AF contains numerous neurotrophic factors secreted by amniotic cells. However, most studies have investigated the protective effects against neuronal degeneration for therapeutic potential. Although AF is circulated into the fetus, its effects on the fetal brain are still unclear. In this study, we showed that AF increased the proliferative properties of fetal neural cells and associated cellular signaling.

Regarding the intracellular signaling underlying the effects of AF, several studies have supported roles for AF in the fetus and adult. In instance, human AF induces the proliferation of fetal and adult skin fibroblasts via ERK and the Akt signaling pathway [22]. Moreover, AF stimulates the Nrf2/Keap1 pathway in forming and repairing epidermal barriers in utero [23]. In this study, we further showed that AF stimulated the MAP kinases pathway, and, in turn, inhibited the activation of GSK-3 in developing neurons. This suppression of GSK-3 ultimately increased the proliferation of neural progenitor cells.

Amniotic fluid is a complex biological material that contains numerous proteins, lipids, even stem cells in the fluid. Using two-dimensional electrophoresis and mass spectrometry, proteome analysis identified 35 proteins in human AF [3]. Moreover, proteomic comparison by gestational age showed large differences in the relative abundances of human AF proteins using two-dimensional electrophoresis [24]. In addition to proteins, cell-free fetal nucleic acids were also detected at much greater concentrations in AF than in maternal plasma [25]. During pregnancy, maternal anxiety caused an increase in stress hormone, corticosteroid levels in AF, as in maternal plasma. Thus, there are many kinds of proteins, lipids, and nucleic acids in AF, which tightly changes in response to various circumstances such as age, stress and diseases states. These materials initiate various intracellular signaling pathways via activation or inhibition of receptors and ion channels, influencing neural development [16, 26]. With numerous biological materials in AF, we should further determine the effective component with regard to neural proliferation in the fetus.

## Conclusions

The AF is originally known to function as a shock absorber to protect against external impacts. The current study further suggest that circulating-AF in the fetus could affect neural progenitor cells via MAP kinases-derived GSK-3 pathway to support fetal brain development. Furthermore, stressful maternal behaviors such as drinking, smoking during pregnancy could increase harmful materials in the AF, which might negatively influence on fetal neurodevelopment.

## Methods

### Amniotic fluid preparation

According to guidelines issued by the Institutional Animal Care and Use Committee at Seoul National University, an embryonic day 16 pregnant Sprague–Dawley (SD) rat was sacrificed in a CO_2_ chamber. The uterus was removed quickly and placed into a 100 mm sterile Petri-dish containing cold Hank’s balanced salt solution (HBSS) on ice. The uterine walls were incised with maintaining the amnion. The amniotic sac was washed three times with cold HBSS, and then we obtained amniotic fluid (AF) in the tube, tearing the amnion using surgical scissors. After centrifuging the AF (5 rpm, 5 min, 4°C), we collected the supernatant fraction.

### Cortical neuron culture

After extracting the AF, we used the embryos to culture cortical neurons. The collected cerebral cortex was transferred into Neurobasal^®^ medium (Gibco), and triturated using a sterile Pasteur pipette. After passing through a 40 μm cell strainer, the cortical cells were cultured on the poly-l-ornithine-coated plates in Neurobasal^®^ medium containing B-27^®^ supplement (Gibco), penicillin/streptomycin (Gibco), and l-glutamine (Gibco) at 37°C in a 95% air/5% CO_2_ incubator.

### Neurosphere formation

To make neurospheres, we first collected the cerebral cortex, as above. The cerebral cortex was placed in DMEM/F12 medium (Gibco), and then triturated using a sterile Pasteur pipette. After passing through a 40 μm cell strainer, the cortical cells were incubated in an un-coated plate in DMEM/F12 medium containing 20 ng FGF, 20 ng FGF, and N2 supplement for 3 or 4 days at 37°C in a 95% air/5% CO_2_ incubator.

### Western blot

Cultured cortical neurons were lysed with RIPA cell lysis buffer (GenDEPOT) containing a protease inhibitor cocktail (Roche). The protein lysates were subjected to a 10% SDS-PAGE gel and transferred to PVDF membrane. The membranes were blocked for 1 h with TBS-T solution (20 mM Tris/HCl, 500 mM NaCl, 0.1% Tween 20) containing 3% skimmed milk powder and then incubated with primary antibodies against ERK (Cell Signaling), GSK-3 antibody sampler kit (Cell Signaling), α-tubulin (Millipore), Nestin (Millipore), GFAP (Sigma), BLBP (Abcam), and Tuj-1 (Abcam) overnight at 4°C on a rotary shaker. Membranes were washed three times in TBS-T solution for 30 min, incubated with secondary antibody for 1 h at RT, and then treated with WEST-ZOL^®^ ECL solution (iNtRON Biotech). Blots were analyzed using an ImageQuant LAS 4000 chemiluminescence system (GE Healthcare).

### Immunocytochemistry

Cortical neurons cultured on round coverslips were fixed with 4% paraformaldehyde. Briefly, primary antibodies raised against Nestin, or Tuj-1 were incubated overnight at 4°C on a rotary shaker. The primary antibody-treated cells were washed three times with phosphate buffer solution containing 0.5% Triton X-100, and then bathed for 1 h at RT with Alexa Fluor 488-conjugated anti-mouse IgG (Invitrogen).

### Flow cytometry

Cortical neurons generated floating neurospheres for 5 days with or without AF. To measure the comparative size of the neurosphere, cells were run through a flow cytometry analyzer, a FACSCalibur (BD bioscience). The sizes of neurosphere were compared through frequencies of R1-gated cells, representing cell populations with FSC-H values above 600.

### Statistics

All results are expressed as means ± SEMs. Student’s t-test was used to determine statistical differences between two means. One-way ANOVA was used to perform multiple comparisons of means followed by the Tukey’s post hoc test. Statistical significance was accepted at *p* values of *<0.05, **<0.01, and ***<0.001, as indicated. All experiments repeated at least three times independent to raise accuracy.
